# Predictiveness and drivers of highly pathogenic avian influenza outbreaks in Europe

**DOI:** 10.1038/s41598-025-04624-x

**Published:** 2025-07-17

**Authors:** Michael Rogo Opata, Andrea Lavarello-Schettini, Jan C. Semenza, Joacim Rocklöv

**Affiliations:** 1 Heidelberg Institute of Global Health, Heidelberg University, Heidelberg, Germany; 2 Interdisciplinary Centre for Scientific Computing, Heidelberg University, Im Neuenheimer Feld 205, 69120 Heidelberg, Germany; 3 Department of Epidemiology and Global Health, Umeå University, Umeå, Sweden

**Keywords:** Avian influenza, HPAI, Machine learning, Climate change, Predictive capability, HPAI drivers, Ecological epidemiology, Climate-change impacts, Influenza virus

## Abstract

Avian Influenza (AI) outbreaks are on an increasing trajectory. This disease carries a substantial economic burden, resulting in considerable losses to farmers with profound impacts on economies. As the outbreaks continue in birds and other unusual host species, further virus evolution and spillover to humans’ risk is anticipated to grow and potentially involve into new pandemics. Despite this, the underlying drivers of the outbreaks remain elusive. We develop machine learning models capable of predicting HPAI events in Europe dynamically uncovering the critical determinants of their onset. Temperature, water index, vegetation index, and poultry density play pivotal roles, with their importance coming into play at different times of the year. Temperature, water index, and vegetation index are important in the ecology of pathogen transmission as well as environmental ecological processes while water index determines how birds aggregate at different locations depending on the season of the year. Combining these drivers, the outbreak pattern is predicted with an accuracy of 94% for model two (M2). A true out of sample with the same model yielded 88% accuracy highlighting its predicting capability. These insights lay a robust foundation for elucidating the intricate landscape of AI outbreaks, offering valuable insights for proactive preventive interventions to mitigate spillover.

**Supplementary Information:**

The online version contains supplementary material available at 10.1038/s41598-025-04624-x.

## Introduction

The contribution of eco-climatic drivers to human disease has been known since time immemorial^1^. This concept also holds true for animal diseases and is fundamental for the understanding of disease emergence and spread. It has become even more apparent in the wake of the recent COVID-19 pandemic which involved the emergence and dispersion of SARS-CoV-2^2^. Other respiratory diseases which pose potential risk of spill-over and spread among human beings are those that are circulating in some poultry populations as of 2023, including H5Nx Goose/Guangdong (Gs/Gd) and H7N9 Anhui1/13^3,4^. There is an increasing concern of such events expanding into a pandemic; therefore, attention has shifted to active surveillance and detection of these outbreaks in several parts of the world involving not only wild and domestic birds, but also increasingly other mammalian hosts^5–9^. The latest major poultry epidemic wave occurred between January 2022 and early 2023 with most outbreaks reported from the Northern Hemisphere^10^.

There are four types of AI viruses namely, type *A, B, C* and D. The H5N1 belonging to type A *Influenza virus* exists in both HPAI and LPAI forms. Its highly pathogenic variant of clade (2.3.4.4b) is known to cause majority of recent global outbreaks and is commonly hosted in ducks, shorebirds, gulls and other waterbirds, naturally *Anseriformes* and *Chadriiformes*^11^. The relationship between these bird hosts and their surrounding ecology plays a critical role in the biology of the disease. The ecological drivers influencing avian influenza include among others, bird host community, migration patterns, and interface between wild birds and poultry^12–14^. Existing evidence alludes to the fact that pathogenic strains are primarily perpetuated through host-to-host transmission during fledging and nesting at water sites^15^. A long-term debate has also existed on how migratory birds contribute to the spread of the virus across regions especially between the Eurasia and Europe^16^.

Sporadic Avian Influenza Virus (AIV) infections in mammals have also been reported since 2003^17^ and have been increasingly reported during the latest poultry outbreak wave in wild terrestrial and aquatic mammals, and more recently, in domestic animals, including pets and other livestock^18–21^. These reports generally coincide with disease events in wild birds as well as poultry and could occur when predating mammals presumably eat infected birds^22,23^. Of course, such a complex mechanism occurs within a shared environment between involved species and is often thought to be driven by various factors including climate variability and change, migration, predatory behavior etc. Since human beings exist in this nexus, the potential spillover of these highly pathogenic viruses is possible considering increased interactions with other susceptible species^24^. In fact, in March 2024, an adult dairy farm worker in Texas, USA, had contact with sick cows with confirmed HPAI A(H5N1) virus infection and subsequently developed conjunctivitis without respiratory symptoms that was confirmed HPAI A(H5N1) virus infection in the conjunctival and nasopharyngeal swab specimens^25^. In May 2024, a confirmed fatal case of human infection with avian influenza A(H5N2) virus was reported from State of Mexico, and while the source of exposure to the virus is currently not known, this virus circulates in poultry (https://www.who.int/emergencies/disease-outbreak-news/item/2024-DON520).

**Fig. 1:**
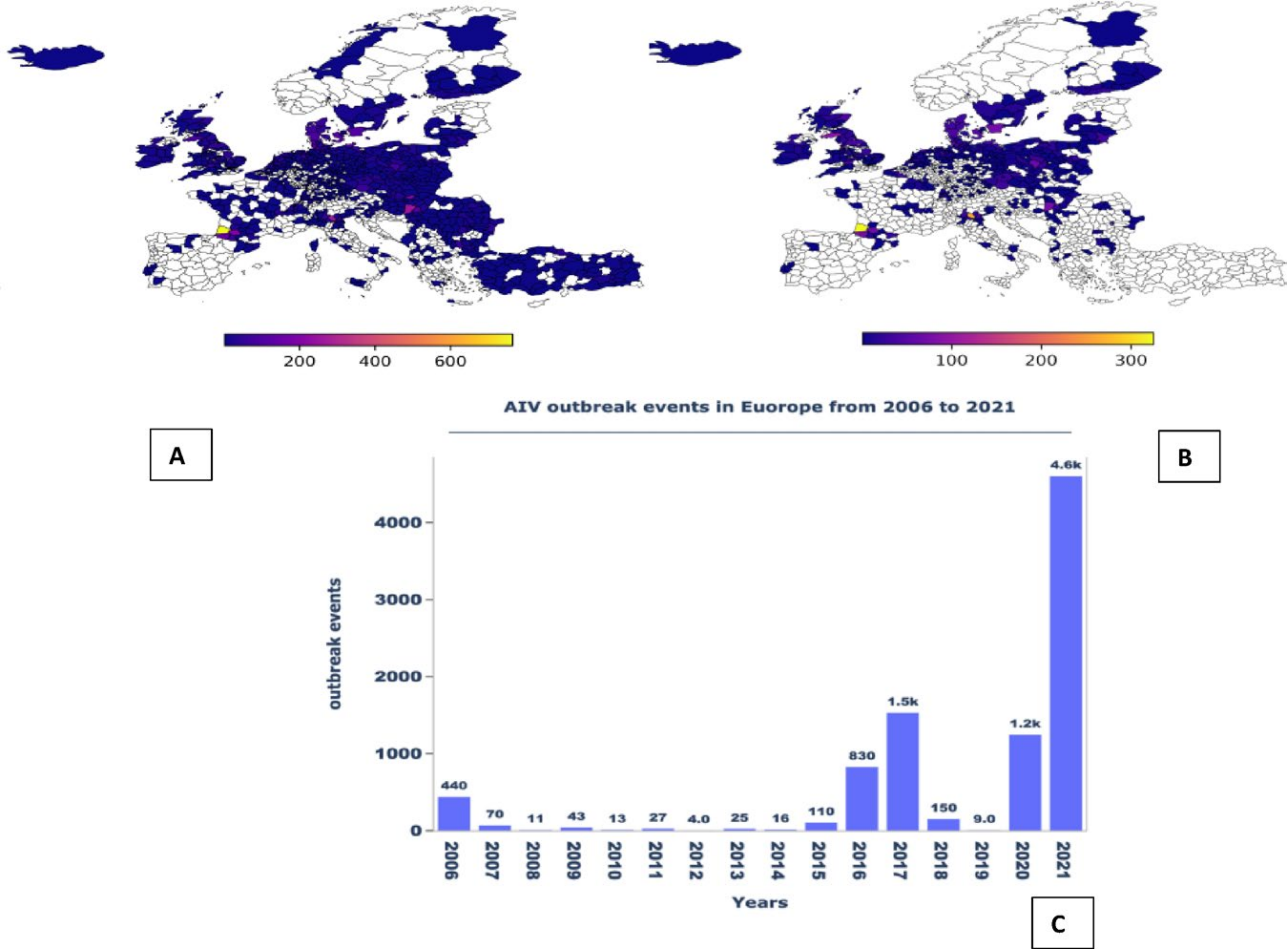
Top left (**A**) shows a timeseries of Avian Influenza outbreaks between the years 2006 to 2021.The top right map (**B**) shows the 2021 AIV outbreak distribution and intensity. (**C**) shows AIV distribution and number of outbreaks in Europe between 2006 and 2021 statistically aggregated at level three of European administration zones. The data consists of poultry, wild birds, and mammals from WOAH (ref = 20).

When AIV outbreaks are detected in animals, Members of the World Organisation for Animal Health (WOAH) shall report them within 24 h of confirmation through the World Animal Health Information System (WAHIS)^10^. This comprehensive database contains useful spatial–temporal information about several animal disease outbreaks captured since 2005 to date. Figure 1 summarizes the European annual AI outbreaks reported through WAHIS between 2006 and 2021. This includes events involving both High and Low Pathogenicity Avian Influenza viruses in poultry and non-poultry birds. There was a significant surge in reported outbreaks between 2016 and 2017. In contrast, 2018 and 2019 witnessed a remarkably low incidence of outbreaks, followed by a sudden and pronounced upswing in cases during 2020 and 2021. The discernible oscillation in these outbreaks underscores the need for a comprehensive study to examine the patterns and potential factors that contribute to the spread of AIVs.

Epidemiology has seen a steady increase in the use of prediction models for early warning systems^26^. For AIV outbreaks, the distance to coastline, distance to wetlands and AIV in wild birds have been used as predictors both on a country scale for Denmark^27^ and European scale^28^. The association between AIV outbreaks and distance to migratory waterfowl sites, distance to major roads and distance to rivers has also been explored in Romania^29^. In California^30^, land cover and distance to coast were predictors of AIV outbreaks. Surface water and domestic bird density were associated with seasonal clustering of AIV outbreaks in poultry in Africa, Europe and Asia using models of landscape epidemiology^31^. Similarly, on a country level, bird density and landscape variables were predictors of AIV outbreaks in the Netherlands^32^.

These tools aid in better understanding of the underlying and sometimes hidden patterns contributing to unexpected outbreaks. If responses are deployed in a timely manner, the adverse economic impact associated with such events could be averted^33^. In the context of AIV, some studies have carried out assessment of outbreaks and the drivers thereof involved in the wild bird-poultry interface^34–36^. To the best of our knowledge, our study is the first to carry out a comprehensive predictive study at a high geo-spatial resolution in Europe.

In this paper, we create explainable and predictive models using machine learning (ML) to study how eco-climatic, social-economic and infected wild birds predict and explain the outbreaks of AIV in poultry and their predictive accuracy. For this, we use data from a variety of sources describing the development of avian influenza in Europe between the years 2006 and 2021. We specifically investigate if eco-climatic and socio-economic variables predict AIV outbreaks as the nature of the association; the importance of time-varying variables change with season and variable interactions; what species of wild birds are most predictive of poultry outbreak events; and, what variable composition provides the most accurate predictions on data withheld from odel fitting.

## Methodology

### Data collection

The data used in this study were obtained from WAHIS. In WAHIS, each event is composed by one or more epidemiologically related outbreak, as assessed by the national veterinary authorities. An outbreak, as defined by the WOAH Terrestrial Animal Health Code, is the occurrence of one or more cases within an epidemiological unit, which in turn can be understood as a group of animals with a defined epidemiological relationship that share approximately the same likelihood of exposure to a pathogenic agent. We included all HPAI outbreaks of domestic and wild birds reported by European countries between 2006 and 2021 (Fig. 1). This is a presence-only dataset, as HPAI has historically been reported by European countries through the Early Warning System of WAHIS. As opposed to other diseases, most countries have an active surveillance approach. Additionally, WOAH’s active search efforts often capture outbreak incidence through unofficial channels (i.e. the media), which are later confirmed or denied by the authorities. A summary of the variant counts in the data is provided in (Fig. SI 1). The point dataset consisting of the region’s longitude and latitude was aggregated at an annual time scale and geocoded into their respective NUTS3 (Nomenclature of territorial units for statistics) regions for mapping onto other variables. (See Table SI1) for the files used to construct the models. Specifically, each outbreak represented by longitude and latitude was preprocessed using python’s geocoder library and consequent variables appended. Since the dataset from WAHIS was a presence-only dataset, there were no missing values to handle. The data was then cleaned by removing points which could not be mapped to specific NUTS3 regions.

Eco-climatic variables i.e., the temperature and rainfall together with high and low vegetation indices were obtained from the Copernicus Climate Change Service (C3S, https://climate.copernicus.eu/the-climate-data-store). The Normalized difference water index (NDWI) and Normalized difference vegetation index (NDVI) were obtained from Landsat 8 Collection Tier 1 and MODIS/Terra Vegetation Indices dataset respectively^37–39^. These variables are known to influence AVI outbreaks by affecting host behavior, habitat suitability, and virus survival and hence relevant to prediction of outbreaks^40–43^. Additional socio-demographic and economic data i.e., the male and female population density, Gross Domestic Production (GDP), and annual trading variables was obtained from the Eurostat database^44^. The trade variables here represent annual exports and imports for each of the NUTS3 regions in goods. The reader is referred to the SI for links to these variables.

Bioclimatic variables (*bio1-bio19*) were included to capture seasonality and complex feature interactions between wild-bird outbreaks and eco-climatic drivers of host-virus amplification and the suitability of geographical introduction (See Table SI2 for details)^45^. Missing values for each of these features were represented by the number (− 999), a format that is internally understood by the XGBoost algorithm. This option was chosen owing to the data sparsity-aware nature of the XGBoost. A further processing of the explanatory features was employed by using wild bird species to predict poultry outbreaks. With these features at hand, we constructed the models to evaluate the accuracy of predicting AIV outbreaks. The dataset was split into three parts. The training data was taken from the year 2006 up to the year 2020. The 2021 data was further split into two parts: validation dataset and test dataset. This split was achieved by randomly selecting data points at a ratio of 80–20 for validation and test respectively. A brief background on the rationale behind data splitting ratios is available in the SI under the methods section.

To test the robustness of the model, we further performed a true out of sample by predicting outbreaks from 2022 and 2023. To adhere to the 80/20 split ratio, data points from 2006 to 2022 (partly) was used to train the model while 2022 (partly) and entire 2023 outbreaks were used for testing. For test data, no random split was employed. Instead, the data was split based on the first regions appearing in 2022 and appended to the training dataset. Consequent data points were set aside for testing. Additionally, sensitivity analysis was performed to adjust for potential reporting bias within WAHIS dataset. For this purpose, four key features were used to generate the weights, namely, NUTS3 region, Year of reporting, poultry density and the reported outbreaks. The weights underwent a further normalization transformation to obtain only positive values necessary for training. Spatial cross-validation was used to adjust for spatial dependency since the outbreaks may be strongly spatially clustered. The NUTS3 id was used as a key indicator of geographically relatedness and groups of these regions were mapped onto the targets (that is, outbreak labels).

### Model assessment, selection, and evaluation

Classification tasks can be carried out using various machine learning algorithms such as logistic regression, classification trees, gradient boosted trees methods, and random forest^46^. We employ the XGBoost algorithm (Python Package version 2.1.4 https://xgboost.readthedocs.io/en/release_3.0.0/python/index.html) which is a subset of the gradient boosted tree family and is known to perform well in general classification studies. The model’s hyperparameters were tuned before construction and the resulting optimal values used for consequent model runs. This process was carried out by setting hyperparameter values of interest within a certain range to scan for optimal combinations that culminated into a robust model. The reader is directed to *Bartz etal* for details on this topic^47^. A tree plot example of the model obtained using hyperparameters is provided in Fig. S2.

Each model was constructed from different engineered features and their targets based on the specific question of interest. Features were categorized into climate, environmental, demographic, economic, bioclimatic, birds, poultry density and trade classes. The climate variables were aggregated into quarters to match the seasonality in Europe, i.e., quarters one (Q1), two (Q2), three (Q3), and four (Q4) correspond to January-March, April-June, July-September, and October-December, respectively. A total of three models were constructed (see Table SI 3). Model 1 (M1) consisted of 50 features and was used to answer a general question; what eco-climatic and socio-economic variables drive AIV outbreaks and how predictive are they? In this model, temperature and rainfall variables for all quarters and other categories were included. In Model 2 (M2), wild infected bird features were introduced additionally (totaling 260 features). Each wild bird row was converted to columns and mapped onto respective positive outbreak label. This transformation acted as a proxy to wild bird presence as opposed to wild bird population, data that was not available at hand. The model was designed to answer the question; how do wild bird outbreaks in combination with eco-climatic and socio-economic drivers affect the prediction of domestic bird outbreaks? In the final model (M3), the wild birds were used as features to predict domestic bird outbreaks while simultaneously disabling their targets. The goal of the last two models was to investigate the role and predictive gain from having wild bird outbreak surveillance data. Furthermore, these two models were used to identify which bird species play a critical role in AIV spread. Biologically, it is known that some strains such as the Gs/Gd HPAI cause severe outbreaks in terrestrial poultry whenever spillover events arise^11,33^. In that respect, we aim to study the on and off influence of this phenomena by tweaking the targets of the wild birds when used as features. The data we used for all the models above consisted of HPAI outbreaks only.

Simultaneous evaluation of the models was carried out using a combination of the *logloss*, the receiver operating characteristic curves (ROC), and estimates of the area under the curve (AUC) evaluation metrics. The respective cross-validation *logloss* and the models’ *logloss* were plotted (Fig. SI 3). While these plots are plausible, interpreting the significance of the models’ results is not straight forward. Local Interpretable Model-Agonistic Explanations (LIME)^48^ and SHapely Additive exPlanations (SHAP)^49^ are two explainable constructs that can be used to complement the interpretation of a ML model. These methods simplify the global complexity in the model and approximate it around the vicinity of instances where the optimization of the resultant model is always treated as a submodular problem. SHAP uses additive feature attribution formulation to break down individual contribution of each feature whose impact is easy to explain. SHAP takes a game theory approach and breaks down individual contributions made by each feature in predicting whether an outbreak is positive or negative. The individual contribution of the drivers was quantified by calculating each feature’s marginal contribution to the model’s output across all possible feature combinations using SHAP. SHAP values are calculated based on Shapely values from cooperative game theory where for each subset of features not including feature $i$, SHAP computes the change in the model’s prediction when $i$ is added. An average of the computation is thereafter performed across all subsets. These values represent the overall contribution of each feature to the model’s capability to correctly predict an outbreak or not. The reader is referred to ref 42 for a detailed explanation of the foundation of SHAP.

XGBoost seamlessly integrates with the SHAP library to provide ranked important features influencing the prediction of a model. Features with high impact on the outbreak prediction were characterized for each constructed model. An aggregation based on individual contributions yielded percentages used to assess the overall important drivers is provided for in the SI. Additionally, key interactions of highly correlated variables highlighting important interplay between wild birds, climate and environment were also analyzed.

### Out of sample predictions

External validation for the model was conducted using 2022 and 2023 outbreak data. The choice of this out of sample confirmed the robustness and predictive capability of the model. In these experiments, the training consisted of outbreaks between the years 2006, 2021, and partly 2022. Here, no random split was conducted. We carefully choose 80 percent of the data until the beginning of 2022. The rest of the data points consisting of majority of 2022 and the entire 2023 outbreaks were used for testing (See Fig. SI 9 for the outbreak distribution). We also performed sensitivity analysis by generating weights on the data to address potential reporting bias within the reported outbreaks in WAHIS.

Spatial correlation problems which may cause data leakage was addressed using spatial cross-validation with NUTS3 region IDs used as the key indicator of spatial correlation. GroupKFold strategy with 5 splits, ensuring that data from the same region was not split across training and validation sets thereby preserving the spatial structure of the data and accounts for potential correlation within the regions was used. Subsequently, the model performance was evaluated on a held-out test set (2022–2023).

## Results

**Fig. 2:**
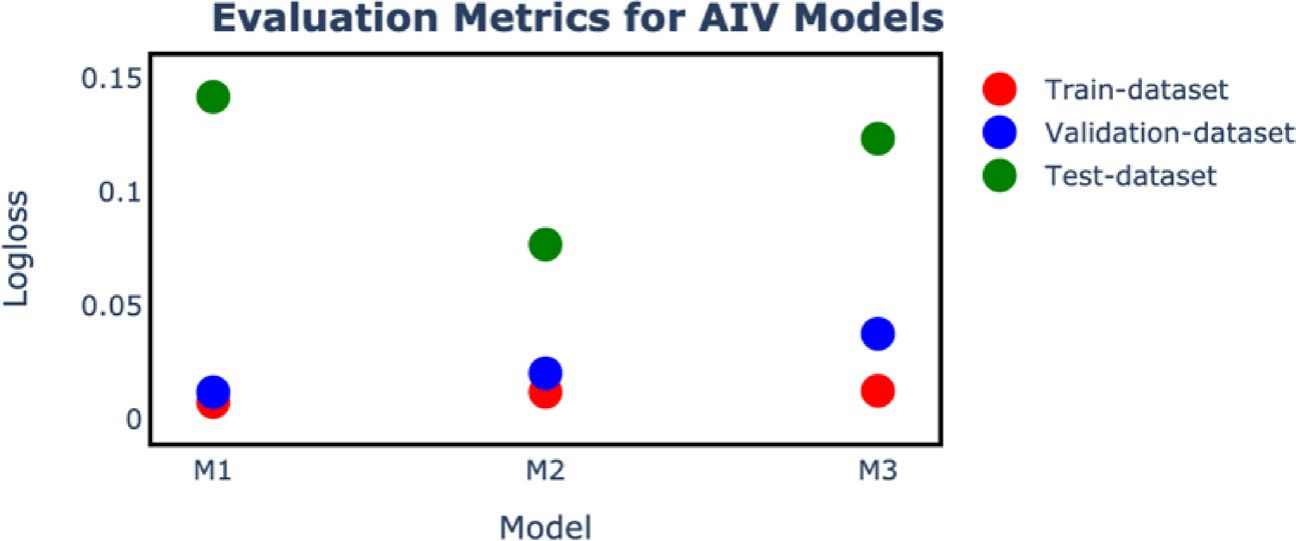
Values of the Logloss Evaluation metric for all models on the validation dataset (2021) and test (2021) dataset. The model M2 performs better compared to M1 and M3 on the test dataset. A separation of the performance on the validation dataset differs as one moves from M1 to M3, while on the test-dataset the minimum is observed for model M2.

We devised a gradient-boosted tree model, based on the XGBoost algorithm, to disentangle the individual contribution of different drivers of AIV outbreaks between 2006 and 2021 in Europe, at a high spatiotemporal resolution. We tested a wide range of explanatory variables of these AIV outbreaks, including climatic, environmental, socio-economic, human population density, poultry density and ornithological variables. The three different models subjected to validation with out-of-sample data revealed high predictive accuracy of the spatiotemporal dynamics of AIV outbreaks among poultry. Based on the logloss evaluation metrics on the test-dataset, M1 had a value of 0.15. On the inclusion of wild bird species as features in M2, the predictive ability of AIV outbreaks was more robust with a drastic shift to 0.08 (Fig. 2). Disabling the wild bird labels on the other hand results in an increase in logloss for M3 to a value of 0.12. Consequently, the AUC scores for M1, M2, and M3 were 0.921, 0.942, and 0.941 at (95% CI) respectively. The true positive rate was 97.55, 97.64 and 98.94 while the true negative rate was 86.79, 90.73 and 89.53 respectively for M1, M2 and M3. M2 therefore emerges to be the optimal model in predicting AIV outbreaks. The above results were obtained using a baseline threshold of 0.5. For brevity, we computed performance metrics at thresholds 0.1, 0.2, 0.3, and 0.4 to gain insights on the extent of specificity and sensitivity changes with threshold shifts (Table SI 4). The outstanding sensitivity and response prediction of M2 led to its choice as our favorable model for result presentation and discussion. It is hereafter referred to as the model. The results and discussions for models M1 and M3 are provided in the Supplementary Information**.**

### Out of sample predictions

Testing the robustness of the model with unseen data resulted in an accuracy of 80% (See the unseen data folder in the review GitHub folder). Including spatial correlation using spatial cross-validation technique resulted in a 1% improvement accuracy i.e. 81% on the test data. We however observed a significant improvement on the model when weights were added. The weighted model resulted in a 88% accuracy on a true out of sample dataset.

### Key drivers of AIV outbreaks

**Fig. 3:**
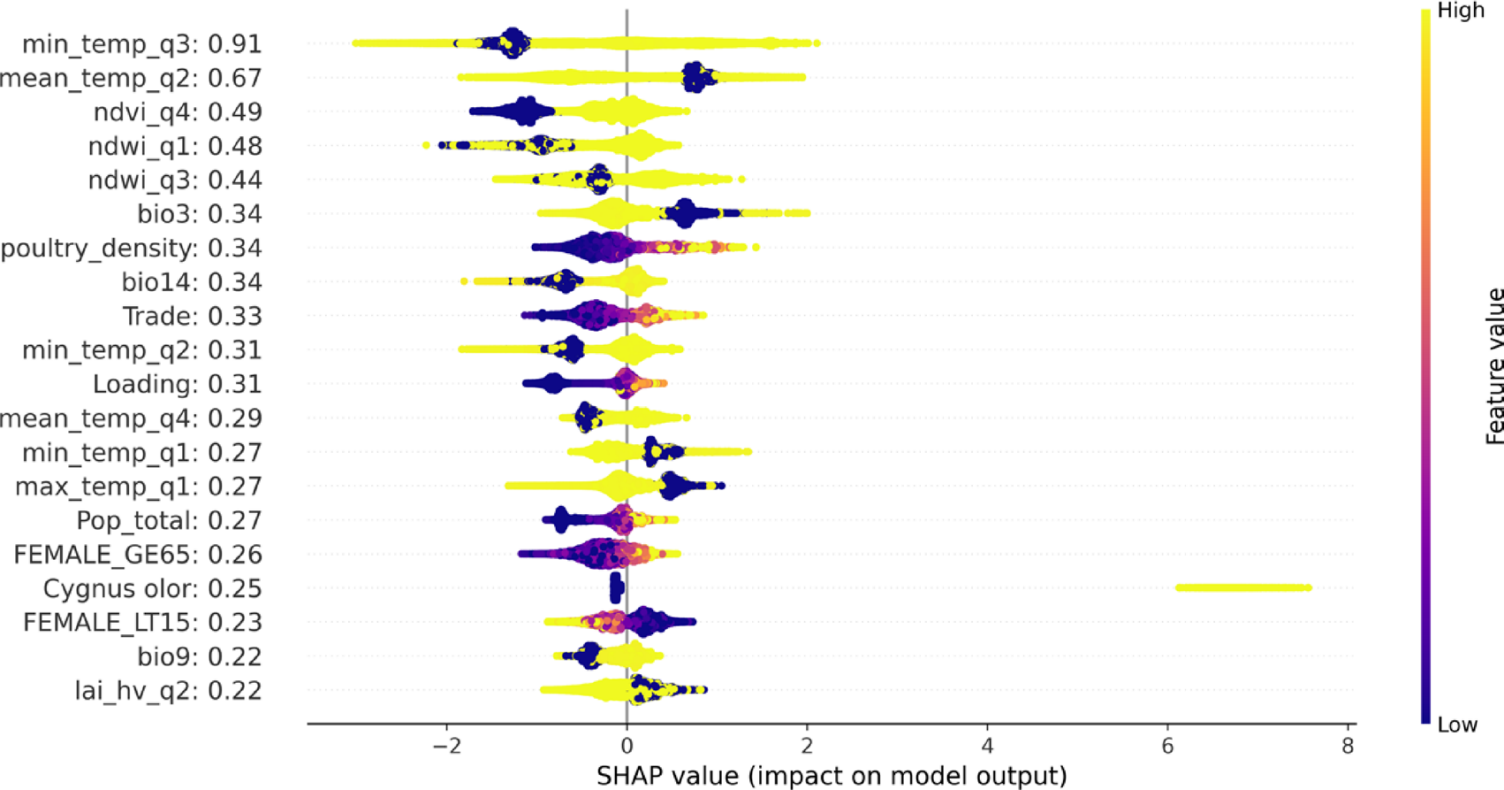
Summary SHAP plot depicting the global as well as the local feature impact on model 2. Temperature, normalized deviation vegetation index, and normalized deviation water index emerge as the top ranking features. Bio6 and wild positively pushes the prediction of the outbreaks with Cygnus olor being the leading species. Low bio6 (temperature seasonality), mean_temp_q2 values has positive impact on the model (+ ve predictions). On the other hand, reduction in water and vegetation in Q4 and Q1 respectively together with low values in min_temp_q3 has negative impact on the model (− ve prediction).

A range of features are found important for predicting AIV in M2 (Fig. 3). Among those, climatic, environmental and vegetation variables rank the highest importance, followed by bioclimatic, poultry density and finally by more general socioeconomic conditions (Trade and population density). The relationship between these variables and the occurrence of outbreaks in poultry is, however, complex and, for example, show non-linear, U-shaped patterns in relationship to temperature. We provide SHAP interactions of these variables with poultry density in the supporting information. In general, as the poultry density increases, SHAP values become positive indicating that high poultry density strongly increases the model’s predicted risk of outbreak (See Fig. SI 8). Additionally, we observed a strong binary spike pattern in the SHap value in relationship to white swan (*Cygnus olor*).

**Fig. 4:**
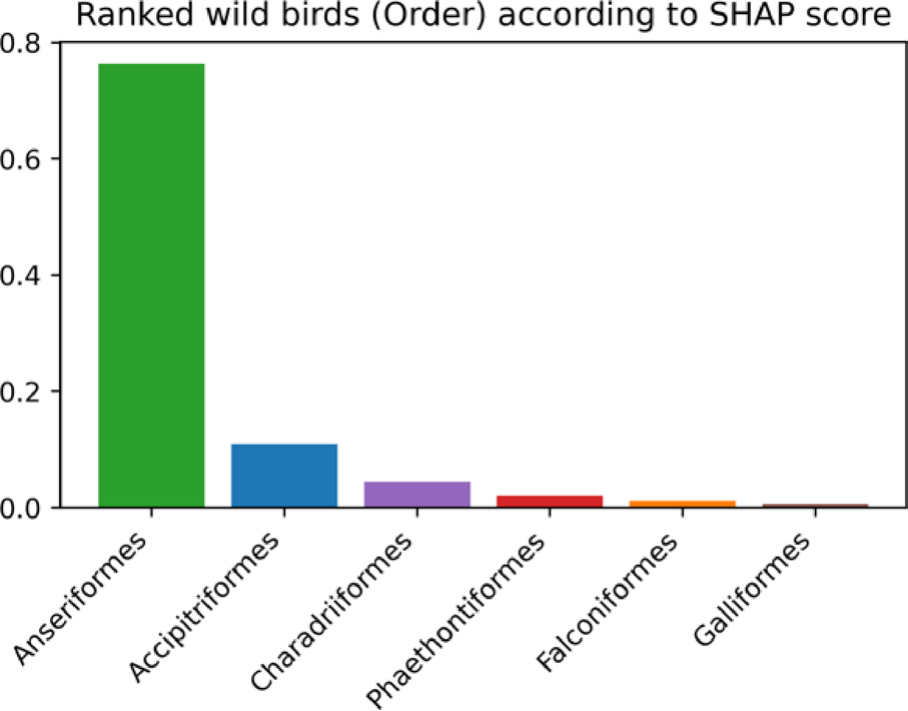
Specific order ranking of wild birds as predicted by M2.

The top global ranking features in the model reveal that cold temperature (min_temp_q3) in the Fall is the most important predictor with a U-shaped pattern where for some regions, the effect of low and high values decrease and increases the prediction. Additionally, the mean temperature (mean_temp_q2) in Spring also plays a critical role. Here, low values have a negative impact on the model. These climate variables might have behavioral implications for the birds but might also affect the environmental fate of the virus, in that colder temperatures are more permissive for viral survival. We capture these possible implications in the form of the minimum temperature of the coldest quarter (min_temp_q1), normalized difference vegetation (ndvi_q4)- and water indices (ndwi_q1). The min_temp_q1 has a positive impact on the model where low temperatures leads to positive label prediction. The low temperatures are known to affect the availability and quality of habitat driving wild birds into denser aggregations increasing likelihood of contact between susceptible and infected ones. During winter, low availability of water as well as vegetation signifies low risk of outbreaks. Both variables play a significant role as natural habitats to the main culprits captured by our model (*Cygnus olor*) which are known to migrate during winter. Wild bird features tend to increase the influence of poultry density and bio3 variables in comparison to M1 (see Fig. SI 4). The cumulative significance of wild bird SHAP values ranks fifth among all the categories for M2. The specific wild bird internal ranking in terms of family order is presented in Fig. 4. Poultry density on the other hand appears among the top ranked variables positively impacting the model. This influence globally ranks after climate and environmental variables. We observe an expected pattern where high values representing dense poultry regions pushes the model towards predicting positive cases while low density pushes the model towards predicting negative cases (see Fig. 3). Detailed plots of the predicted regions captured by our model compared to the poultry density within the respective regions can be found in (Fig. SI 7). In general, temperature, water index, vegetation index, poultry density and bio3 play a critical role in HPAI influenza outbreaks according to our model. The relationship varies across seasons during the year but is dominant in the first and third quarters for the top-ranking variables.

**Fig. 5:**
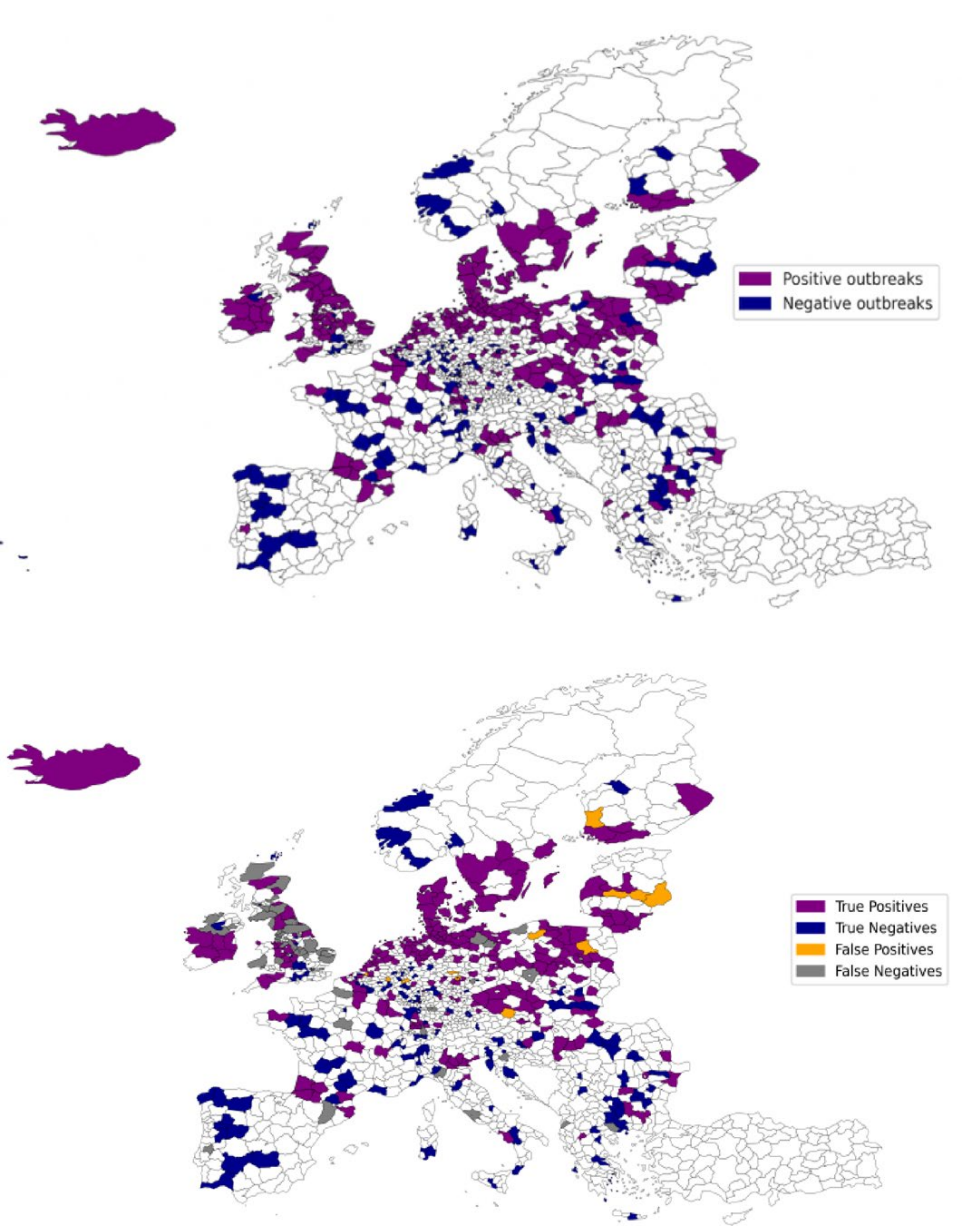
Test dataset labeled positive and negative used in testing the model on data from 2021 (Left). Predicted AIV outbreaks binned into True positives (Actual positive outbreaks predicted), True negatives (Actual negative outbreaks predicted), False positives (Negative outbreaks predicted as positives) and False negatives (Positive outbreaks predicted as negatives) to the right based on the test data from 2021.

An overview of the categories consisting of aggregated features derived from SHAP values (Fig. SI 5) shows climate ranks as a top influencer of AIV outbreaks, followed by environment and bioclimatic variables respectively. We provide the distribution of outbreaks for 2021 test data and the predicted distribution in Fig. 5 with the overall accuracy of 94%. These figures highlight the ground truth data against the color coded predicted True Positives (TP), True Negatives (TN), False Positives (FP), and False Negatives (FN).

### Feature interactions

Bi-variate interaction analysis indicates that in the model prediction, poultry density as well as birds’ species positively interacts with temperature seasonality (bio15, bio3, and bio4) respectively when predicting poultry outbreaks (Fig. SI 6). Among the bird’s species, *Cygnus olor,* belonging to the family Anatidae and Order Anseriformes has the highest local impact with the largest positive SHAP value on a subset of the models’ data. Accipitriformes and birds of unknown species within the family Anatidae (Order Anseriformes) as well as the Laridae (Charadriiformes) rank second and third respectively according to the SHAP scores (Fig. 4).

## Discussion

AIV is not only a threat to food security and bird welfare but also to human health. The ability of AIV to cross the species barrier and to infect humans raises concerns about their epidemic and pandemic potential. Our investigation focused on studying the drivers and predicting the dynamics of the spread in poultry. We included wild birds as features to investigate their influence in predicting these poultry outbreaks. Such outbreaks can be indicative of human spillover risk as poultry are in close contact with humans. Additionally, public health measures to prevent the spread of AIV also rely on monitoring and surveillance of wild and domestic birds^27^. It entails monitoring sentinel birds in the wild or in a commercial poultry flock, diagnostic testing, and surveillance of flock mortality and morbidity^50,51^. AIV detection can then trigger a range of containment efforts, including enhanced biosecurity at poultry farms, movement ban, vaccination and culling of the entire flock and other flocks around the affected farm, to prevent further dispersion^52,53^. The enactment of these measures often depends on the timing of the alert which in turn depends on the sensitivity of the surveillance system.

Our findings elucidate the contextual determinants of AIV transmission in Europe and rank the importance of specific drivers of the reported AIV outbreaks analyzed in this study. We define the climatic and environmental conditions under which these outbreaks occur, namely the colder temperatures in the Fall and the importance of vegetation and water features as well as densely populated farming regions. We also disentangle the contribution of wild birds to AIV outbreaks and other socioeconomic indicators, such as trade of goods. These findings are important considering AIV surveillance efforts that often lack sensitivity and specificity. Our findings can help target sentinel surveillance to improve the ascertainment proportion of cases and advance early warning systems for AIV outbreaks.

The three most important variables that positively impact our models’ prediction are the poultry density, mean temperature for quarter 3 and environment (water and vegetation indices) (See Fig. 3). This relationship reveals dominant drivers of AIV outbreaks as predicted by our model. These key predictors can be integrated into surveillance and intervention strategies with the insights applied in practice where local authorities could implement risk-based surveillance. This can be achieved by combining dynamic poultry density maps, temperature trends, and satellite environmental data to create predictive maps. These maps would then inform targeted biosecurity and contingency planning such as ring vaccination or pre-emptive vaccination for poultry within high-risk zones. Additionally, climate-informed intervention timing such as movement restrictions or awareness campaigns during Q3 can be implemented. For example, farm infrastructure can be adapted in line with seasonal climate trends to reduce virus susceptibility and bird stress, factors known to play important roles in the spread of AIV. Poultry in high-density farms are often more susceptible to diseases due to weaker immune systems. Factors contributing to this include limited exposure to diverse pathogens, which can impede the development of robust immunity, and stressors associated with overcrowding, such as social stress and reduced feed intake. These stressors have been shown to impair immune responses, increasing disease susceptibility.

We equally observed a strong local effect in wild birds with *Cygnus olor* emerging as the main species with a strong positive impact. The lowest temperature of the coldest month (min_temp_q1) also has a positive impact on the predictions, where low values result in higher AIV outbreak predictions. Indeed, AIVs tend to survive longer in colder ambient conditions, as lower temperatures facilitate their viability outside the host^54^. This might be one of the reasons why most AIV outbreaks have been historically reported during the winter season in the Northern Hemisphere. Conversely, an increase in the maximum temperature in quarter two negatively impacts the prediction. In quarter one, a decrease in the water index results in a low likelihood of outbreaks while an increase is observed in the second quarter. A similar effect is observed for the normalized vegetation index (NDVI) in quarter four where low vegetation index results in low prediction rates. This model therefore suggests that AIV outbreaks seem to be driven by temperature changes between the first and the second quarters of the year when migrating birds gather at environmental sites, and increase their contact rates, possibly during feeding, mating or communal roosts. Thus, for most datapoints, AIV outbreaks occur at low temperatures in the first and second quarters of the year.

*Cygnus olor*, the top-ranking bird species implicated in AIV outbreaks in our model, interacts with temperature seasonality (bio4), a bioclimatic variable (See Fig. SI 6). Since bio4 is known to span several years, the influence of *Cygnus olor* seems to cut across the four quarters as temperatures vary. Coupled with the above findings, our results highlight the intricate interplay between wild birds, climate, and environment. Previously, it has been argued that AIV spread was mainly due to human and trade activities dismissing the role of wild birds. However, wild birds have been implicated in the spread of AIV in remote areas^55^. Studies have also found positivity rate for AIV in Italy with notable seasonal variations underscoring the role of migratory waterfowl in spread of AIV^56^. HPAIV infections with H5N1 among mortalities in mute swans (*Cygnus olor*) have been documented in Germany in part due to their large size and ease of identification^57^. The reporting region, known to be a temporary resting site for migratory birds as well as other mammals, further highlights the importance of congregation at certain sites. Similar observations have been reported in other regions including East coast Canada with incidences in both poultry and wild birds^58^. A critical importance is the bird-environment and avian influenza relationship to both wild bird population and human health in Europe. Extreme weather events that affect the ecology and demography of wild birds have been found to influence the development and spread of avian influenza^59,60^. The human health implications for the AIV spread in the wider Europe continue to raise health concerns especially when workers interact with infected birds, posing potential zoonotic transmission risks.

Highly pathogenic avian influenza (HPAI) outbreaks in remote areas tend to be considered rare events since normal occurrences are restricted to areas adjacent to poultry farms. Indeed, it has been found that HPAIV wild bird reporting increases in the vicinity of poultry farms. This is due to genetic proximity to poultry which pose a wider risk of infections. *Beerens etal* details the genetic traits of wild birds suspected to introduce several poultry H5N6 infections in the Netherlands with *Cygnus Olor* and *Anatidae* being among the culprits^61^. Specifically, the genetic analysis showed that poultry outbreaks were traced to contact with wild birds rather than poultry-to-poultry transmission. Other studies have suggested observation in poultry infection spikes which were preceded by detections on wild birds for both the years 2014 and 2017 in Europe^62–64^. A great deal of these cases has been concluded to be caused by biological and ecological factors. Other studies have also used epidemiologic and genetic data to disentangle transmission dynamics of HPAIV^65^. Collectively, the genetic analyses demonstrated identification of wild birds as the primary source of HPAI outbreaks in poultry for some years underscoring the need for integrated surveillance that consider both wild and domestic avian populations.

A counter threat to wild birds from poultry infections has also been reported in literature. Proximity to poultry as well as humans often leads to such significant infection spikes posing existential threats to local biodiversity^66^. Our model shows significant poultry density information gain when wild birds are added as features suggesting an existing relationship. However, this information does not necessarily mean that they are implicated in every poultry outbreak especially in variants other than H5. Nevertheless, recent evidence points towards increasing correlation in the role played by wild birds and poultry in the spread of H5 HPAI virus^67,68^. Switching the wild birds’ labels drives the poultry density global SHAP ranking even higher (See Fig. SI 4) highlighting the critical role played by high density farms in the spread of HPAI in the absence of wild bird cases. M3 is explored further in the SI discussion section. In terms of accurately capturing these high-density farms, our model performs well (see Fig. SI 7). The population density regions that positively impact the model correlate with the actual concentration of poultry head counts in the respective farms confirming the importance of this variable. In other words, our model predicts farm-to-farm transmission likelihood within the high-density regions. While this may be obvious, such a model could be used to gauge the direction of spread given initial farm infections and mitigations developed. Once the direction of spread has been determined, appropriate intervention measures can be taken. For example, neighboring localities may be notified, harmonizing movement controls thereby establishing zonal restrictions. Constant monitoring protocols may be created such as temporary notification system within a certain radius, say 30 or 50 km. Sampling frequency and environmental swabbing can also be intensified in high-risk zones by expanding active surveillance in farms and known wild bird habitats along predicted spread paths.

There however exist low SHAP importance of socioeconomic variables such as human density, trade, and GDP. This suggests potential disconnects between their practical utility and theoretical relevance. One possibility is the spatial or temporal misalignment between disease events and socioeconomic indicators. Another possibility is the presence of collinearity with stronger predictors such as poultry density or surveillance intensity, causing their marginal contributions to be suppressed in tree-based models. One may speculate that these features may influence disease emergence in indirect or threshold-based ways that require nonlinear transformation or interaction modeling to capture as previously reported^69^.

These glaring facts call for the need to build pandemic preparedness tools using a One Health framework, as well as strengthening early warning systems. Improving both active and passive surveillance systems by collecting and analyzing data to mitigate risks is one approach^70^. Another, more cost-effective method would involve simulation and modeling of existing data to predict outbreaks. In both cases, high success rates depend on reliable strategies to collate data regarding the susceptible wild bird populations, a task that is always difficult to achieve^71^. Ideally, a mix of both methods would result in a holistic surveillance approach.

We believe that this current work takes a step towards achieving this mean feat. Using a high-resolution dataset and robust models, we identify important nexus contributing to HPAI AIV outbreaks. Our model delineates the climatic, environmental, socioeconomic, and biotic conditions that favor AIV outbreaks. These insights can inform the timing and geographic expanse of active or passive surveillance of wild birds and poultry flocks^27^. It can also direct the spatiotemporal extent of environmental sampling^72^ and citizen science^73^ initiatives to advance the sensitivity of AIV surveillance. The combination of our modeling approach with targeted data collection can further map high risk areas and periods of the year that are prone to AIV outbreaks. Such continuous data collection ensures accurate capturing of complex information enabling quick interventions whenever outbreaks arise.

Practical implications of our model include scalable deployment and real-time monitoring of key indicators allowing for rapid adaptation to new data and changes in environmental conditions such as effect on temperature shifts to bird migration patterns. New predictions can therefore be generated in near-real time hence identifying high-risk regions and high-priority farms and resource allocation to these areas. Such targeted monitoring ensures efficient resource allocation avoiding blanket surveillance and interventions across all farms. Furthermore, the models can be used for early detection and containment efforts before they spread widely, thereby preventing large-scale economic losses, containment measures that undermine animal welfare, and public health impacts. For example, in climate change scenarios, where several conditions constantly shift, the models can be adaptively trained with availability of more data improving over time hence incorporating new patterns, features, and ecological changes affecting AIV dynamics. We believe that such applications could improve current measures in handling AIV outbreaks.

## Limitation and outlook

We present here a base model which identified the main drivers of AIV outbreaks in Europe. However, there exist some shortcomings of our model. The dataset does not differentiate between the different surveillance systems that are employed in European countries. It does not account for differences in HPAI AIV detection strategies, sample collection efforts, or intervention measures. Moreover, the model does not exhaustively highlight the complexity of AIV viral sharing between wild birds and poultry, as they are not equally susceptible to infection. Domestic birds are likely to be more at risk of infection from wild birds than the reverse. Factors such as virus clades have not been captured in the model. This is due to the limitation in reporting information of the outbreaks in the data we have. For example, some cases are reported as HPAI, but the specific clade information was missing (See Fig. SI 1).

There is therefore the need to include complete variant information together with their respective clade/lineages to capture complete mechanistic picture. Since this information is being tracked by WOAH as of 2022, refined studies using these new datapoints are needed to understand how climate and environment change contribute to the variant evolution and the spread of Avian Influenza.

Recently, there have been reported sporadic human AIV events which are thought to originate from low AI pathogenic pathways. A more complete picture can be achieved by overlaying existing animal AI data with human AI data to identify existential threats of avian outbreaks and consequent mitigation strategies. Including several features related to the susceptible animal population such as wild bird density, wild bird species distribution, and migratory patterns, trade of animals and animal products, among others, as predictors of AI outbreaks could also enhance prediction. Utilization of the so-called hybrid models (a combination of ML models with physics aware models) to investigate dynamic roles played by additional variables such as wind speed may also contribute towards understanding the animal-human disease interface.

## Conclusion

In this work, we have provided the key drivers of HPAI outbreaks at the European NUTS3 level. Supervised machine learning as implemented in the XGBoost software package generated the base model with high predictive ability. For the interpretation we used SHAP, a game theory engine, which provides an easy way to explain the underlying complexity of the predictors.

Specifically, poultry density, climate and environment are predicted to be the key drivers of AIV outbreaks in Europe. These three drivers serve as main indicators that can aid in identifying patterns from animal data for continual monitoring of potential AIV outbreaks. The key climate indicators implicated in driving AIV outbreaks are the temperature of the coldest month, the mean temperature of quarter two and the minimum temperature of quarter three. Water (quarter one) and vegetation (quarter four) indices also play a critical role. In some regions, wild birds, especially *Cygnus olor* have the highest impact on our model, a finding that resonates with literature reports for the case of H5 variant.

This work has therefore highlighted various patterns and variables that can be used for sentinel surveillance to monitor AIV outbreaks and for creating early warning systems. Our work lays a foundation in the quest to integrate animal, human, and eco-climate data to create pandemic preparedness systems. Such complex data could help in creating strong surveillance tools to capture potential risks posed by changing climate patterns, wild bird migratory patterns and re-infection cases. Additionally, complex methods of collating data as well as continuous analysis would result in reliable risk assessments and foresightful policy developments.

## Electronic supplementary material

Below is the link to the electronic supplementary material.

Supplementary Material 1

## Data Availability

The data and code used in this study is available on GitHub and can be accessed on the web here https://github.com/rogomichael/onehealth_data The specific links directing the reader to the data used are provided in the supplementary information document.
