# Supplementary material for: Predictiveness and drivers of highly pathogenic avian influenza outbreaks in Europe
Source: Sci Rep. 2025 Jul 17;15:20286. doi: 10.1038/s41598-025-04624-x (PMC12271378; doi:10.1038/s41598-025-04624-x)
Supplement: Supplementary file 1 — Supplementary Material 1 [file 41598_2025_4624_MOESM1_ESM.docx]

Predictiveness and Drivers of Avian Influenza in Europe

*Michael Rogo Opata^1,2^ Andrea Lavarello Schettini^1,4^, Jan C. Semenza^1^, Joacim Rocklöv^1,2,3^**
*Interdisciplinary Center for Scientific Computing, Heidelberg University, Im Neuenheimer Feld 205, D-69120 Heidelberg, Germany.*

**Corresponding author. E-mail :* [*joacim.rockloev@iwr.uni-heidelberg.de*](mailto:joacim.rockloev@iwr.uni-heidelberg.de)*, Heidelberg University, Im Neuenheimer Feld 205, 69120 Heidelberg, Germany*

*1. Heidelberg Institute of Global Health, Heidelberg University, Heidelberg, Germany*

*2. Interdisciplinary Centre for Scientific Computing, Heidelberg University, Heidelberg, Germany*

*3. Department of Public Health and Clinical Medicine, Section of Sustainable Health, Umeå University, Sweden*

*4. World Organisation for Animal Health (WOAH), Paris, France*

**SUPPLEMENTARY INFORMATION**

# **Data Collection**

The outbreak data used in the study was obtained from WAHIS. All the 183 WOAH Members, through their veterinary authorities, are required to submit an immediate notification anytime an exceptional epidemiological event concerning high impact animal diseases (WOAH-Listed diseases) unfolds in their territory. This immediate notification must be followed by weekly follow-up reports until the event is resolved, the disease is eradicated or, until the disease is considered established and endemic in the country. An exceptional epidemiological event refers to any introduction or recurrence of WOAH-Listed diseases into the country’s territory.  A complete description of the variables is shown in (Table SI 2).

| A  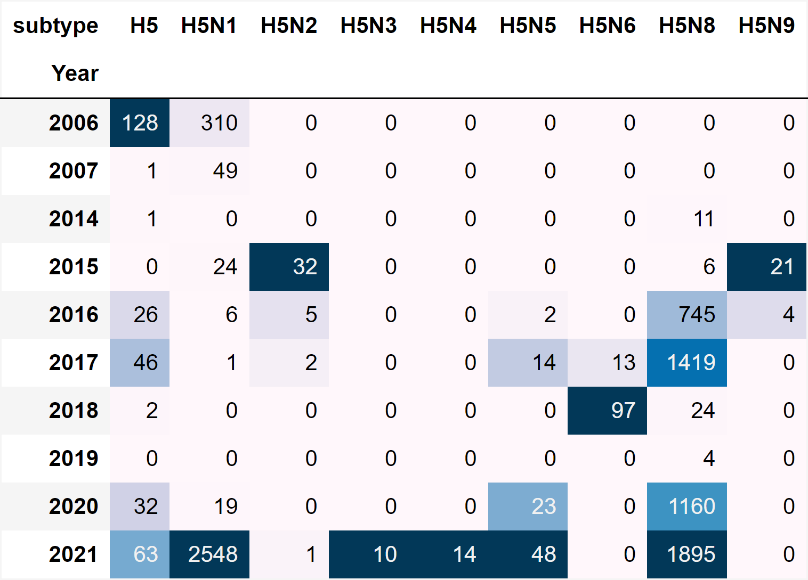 |
| --- |
|  |
| B  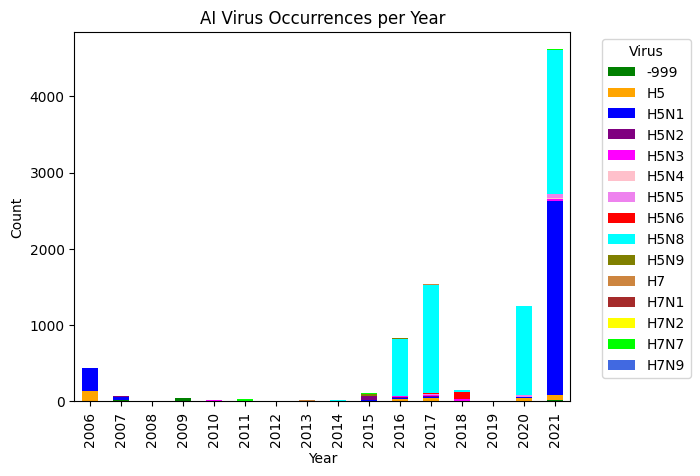 |

**Figure SI 1**: (**A**) Specific AI variant counts as obtained from WAHIS Bar graph depicting specific variants. (**B**) We only study HPAI outbreaks as LPAI variants are very few in number. Missing classifications are represented as -999 in this table as well as in the model.

# Data Availability

The data and code used in this study is available on GitHub and can be accessed on the web here <https://github.com/rogomichael/onehealth_data.git>

| Poultry density data | <https://ec.europa.eu/eurostat/databrowser/view/ef_lsk_poultry/default/table?lang=en&category=agr.ef.ef_livestock> |
| --- | --- |
| Climate data | <https://cds.climate.copernicus.eu/cdsapp#!/dataset/insitu-gridded-observations-europe?tab=overview> |
| Environmental data | <https://cds.climate.copernicus.eu/cdsapp#!/dataset/reanalysis-era5-land-monthly-means?tab=overview> |
| Bioclima data | <https://www.worldclim.org/data/monthlywth.html> |
| Shapefile | <https://ec.europa.eu/eurostat/web/gisco/geodata/reference-data/administrative-units-statistical-units/nuts#nuts21> |
| Trade data | <https://ec.europa.eu/eurostat/databrowser/view/road_go_na_ru3g/default/table?lang=en> |
| Economic data | <https://ec.europa.eu/eurostat/databrowser/view/nama_10r_3gdp/default/table?lang=en> |
| Demographic data | <https://ec.europa.eu/eurostat/databrowser/view/demo_r_pjanaggr3/default/table?lang=en> |
| Avian Influenza Virus data | <https://wahis.woah.org/#/home> |

**Supplementary Files**

The files provided in this section can be used to reproduce the results reported in the main article. The description provides a brief explanation of the contents and the purpose they served.

| **Table SI 1**: Table of supplementary files used for constructing the AIV models | | |
| --- | --- | --- |
|  | **File** | **Description** |
| 1. | ranked_wild_bird_species.xlsx | Contains ranked wild bird score according to their respective impacts on the model as generated from SHAP score. |
| 2. | m1train.xlsx  m1test.xlsx | Training and test dataset for Model 1 |
| 3. | m2train.xlsx  m2test.xlsx | Training and test dataset for Model 2 |
| 4. | m3train.xlsx  m3test.xlsx | Training and test dataset for Model 3 |

| **Table SI 2**: Spatial-temporal features and targets used in constructing AIV models for the years 2006 to 2021. The features were engineered based on the question answered by the model. | | |
| --- | --- | --- |
| Feature category | Target variables | Target names |
| Environmental | Vegetation index | lai_hv_* = high vegetation index |
|  |  | lai_lv_* = low vegetation index |
|  | Normalized deviation water index | ndwi_* |
|  | Normalized deviation vegetation index | ndvi_* |
| Climate | Temperature | min_temp_*  mean_temp_*  maximum_temp_* |
|  | Precipitation | total_rain_*  total_rain_*  total_rain_* |
| Bioclimatic | Temperature-related | BIO1 = Annual Mean Temperature  BIO2 = Mean Diurnal Range (Mean of monthly (max temp - min temp))  BIO3 = Isothermality (BIO2/BIO7) (×100)  BIO4 = Temperature Seasonality (standard deviation ×100)  BIO5 = Max Temperature of Warmest Month  BIO6 = Min Temperature of Coldest Month  BIO7 = Temperature Annual Range (BIO5-BIO6)  BIO8 = Mean Temperature of Wettest Quarter  BIO9 = Mean Temperature of Driest Quarter  BIO10 = Mean Temperature of Warmest Quarter  BIO11 = Mean Temperature of Coldest Quarter |
|  | Precipitation-related | BIO12 = Annual Precipitation  BIO13 = Precipitation of Wettest Month  BIO14 = Precipitation of Driest Month  BIO15 = Precipitation Seasonality (Coefficient of Variation)  BIO16 = Precipitation of Wettest Quarter  BIO17 = Precipitation of Driest Quarter  BIO18 = Precipitation of Warmest Quarter  BIO19 = Precipitation of Coldest Quarter |
| Trade | Goods traded/year | Loading = Annual traded goods (Exports by each statistical region)  Unloading = Annual trading goods (Imports by each statistical region) |
| Economic | Income/year (in Million Euros) | GDP_Annual = Annual mean regional income |
| Demographic | Female population /year | F_TOTAL = Total Female population |
|  |  | F_Y_15-64 = Female 15 – 64 Years |
|  |  | F_Y_GE65 = Female > 65 Years |
|  |  | F_Y_LT15 = Female < 15 years |
|  | Male population / year | M_TOTAL = Total Male population |
|  |  | M_Y_15-64 = Male 15 – 64 Years |
|  |  | M_Y_GE65 = Male > 65 Years |
|  |  | M_Y_LT15 = Male < 15 years |
| Birds | Wild bird population | Wild bird species excluding other mammals |
| Avian Influenza outbreak events | Response variable | Label = NUTS3 Avian Influenza virus outbreak events** |

** The respective features were aggregated at quarterly intervals to represent the seasonality in Europe. Each value constitutes q1, q2, q3, and q4 variables. ** Labels were also manipulated by switching them on and off to investigate phenomena of interest.*

# Methods

Models were tuned to generalize well on new test data. In general, at each node, the ‘learnable’ variables are the choice of decision. We therefore use them to set numeric thresholds that help in informing why we take the left or the right branch. In other words, our models utilize tree-based methods analogous to a normal tree as shown in Figure SI 2 . We make decisions based on the weights of each branch. Ideally the algorithms can be made more conservative or non-conservative through these values. The set of the hyperparameters we use in this work can be broadly explained as:

1. Eta (learning rate): Used to shrink the weights on each step hence making the model more robust.
2. scale_pos_weight: Used for scaling unbalanced data for faster convergence.
3. num_boost_round: Represents the number of rounds for boosting.
4. max-depth: Controls overfitting by preventing learning relations very specific to a particular sample.
5. gamma: Makes the model more conservative in the sense that a node is split only when the resulting split gives a positive reduction in the loss function.
6. subsample: Gives the fraction of observations to be random samples for each tree.
7. colsample_bytree: Represents the fraction of columns to be random samples for each tree.
8. min_child_weight: Used to control overfitting in the sense that higher values restrict the model from learning relations that are specific to the sample of a selected tree.
9. reg_alpha: represents L1 regularization term on weights.
10. reg_lambda: represents L2 regularization term on weights.


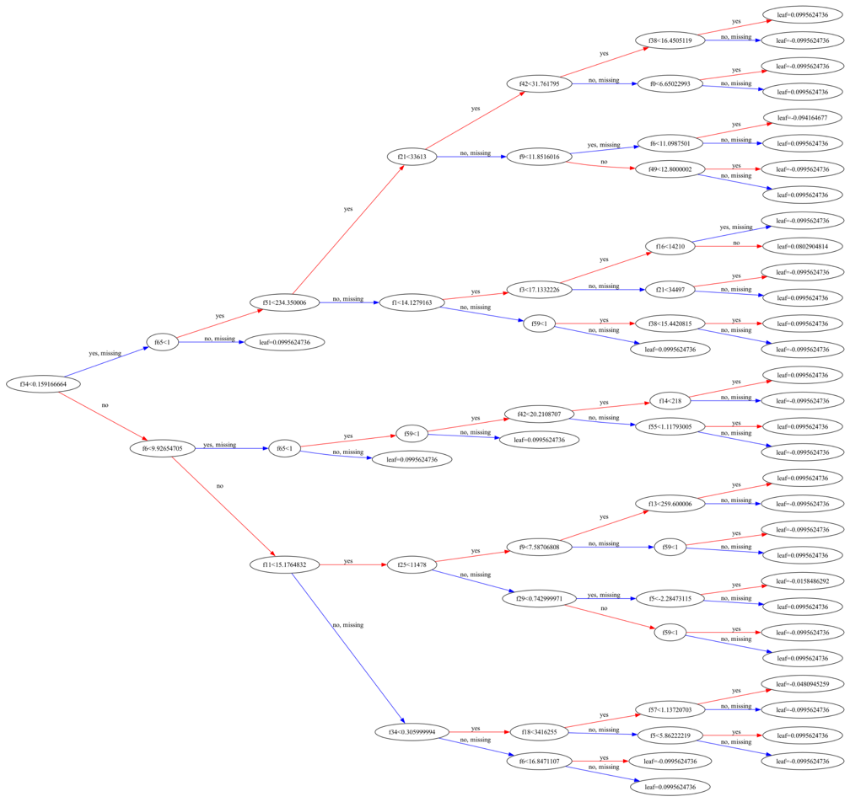


**Figure SI 2**: Tree plot example of M2 explained above.

We employ the python version of XGBoost with a combination of the scikit-learn API [1]. The algorithm has previously been tested to be superior to support vector machine (SVM), random forest, näive model, and lightGBM (light gradient boosted method) in a similar study at NUTS3 level for the West Nile Virus (WNV) [2]. The main advantage XGBoost algorithm has over other methods is its novel sparsity-aware and weighted quantile nature which enables handling of huge dataset that can fit into memory [3]. In the context of our current study, classification trees can be used to provide prediction of dependent classes such as AIV presence versus AIV absence as influenced by the given independent variables. Since our data has been geocoded into specific regions, a regression mathematical relationship for predicting the outbreak events was derived. The logistic regression task involved splitting nodes and pruning trees thus one can represent a region $R_{m}$ with $N_{m}$ observations in node $m$ as:

$$\hat{q}_{mk}=\frac{1}{N_{m}}\sum_{x_{i}\in R_{m}} I\left( y_{i}=k \right)$$

Here the features space has been partitioned for each region m. Typically, the training data is numerically represented as $\boldsymbol{x}_{i}$ and their respective targets $\boldsymbol{y}_{\boldsymbol{i}}$; where $\boldsymbol{y}_{\boldsymbol{i}}\boldsymbol{\in0,1}$ and is equivalent to $\boldsymbol{k}$ in the above equation. This constitutes the basis of the so-called objective function (training loss and regularization terms) [4]. The XGBoost internal workings classify a tree model as

$$\hat{y_{i}}=\sum_{k=1}^{K} f_{k}\left( x_{i} \right),f_{k}\in\mathcal{F}$$

Where $\boldsymbol{K}$ is the number of trees and $f_{k}$ is a function which determines, for each iteration, the trees generated in the functional space $\mathcal{F}$. An objective function such as this one can then be optimized to produce an efficient generalized model. In principle, we combine many ‘weak’ learners/classifiers to produce better learning trees than using a single classifier [5]. This is achieved by greedily adding trees that mostly improve the model using second-order optimization approximations. See ref [3] for further details of XGBoost equations and derivations. We equally performed cross-validation studies using XGBoost internal k-fold strategy. Finally, we construct our models with the resulting tuned parameters.

## Train-test data split ratios

Different structures can be used to divide data under study for the purposes of training and consequently testing the output of interest. A common strategy is to use a split ratio of twenty/eighty percent (20/80) for the test and train sets or thirty/seventy percent (30/70) option [4]. This ratio has been studied to work in several approaches including bootstrapping and is known to optimize performance [6]. Ideally, one can further split the data into three sets (Train, validation, and test) in cases where there exist enough data points. The validation set in this case serves a purpose like the cross-validation approach in cases where data is trained and tested in portions at the same time. The most common cross-validation is the *k-fold* or stratified-*KFold* [7]. This approach ensures that the model is not overfitted when learning the labels of interest.

Our data was split in to three parts (train, test, validation) due to the large samples in the year 2021 which were more than half of the positive labels. This approach ensured that our model does not suffer from overfitting during training. Furthermore, we balanced our labels by scaling the positive and negative classes using the inbuilt XGBoost weight scaling feature.

# Results

**Evaluation metrics**

The optimized parameters for each of the models are summarized in *Table SI 3* for reproducibility purposes. The respective graphs of the training versus the cross-validation experiments are also shown in Figure SI 3. A convergence trend is observed in the optimization steps signaling stability of the models. Consequently, the performances of the models on the test dataset were evaluated using these parameters.

| ***Table SI 3****: Optimal values for each of the models we constructed. Respective features in the models were engineered hence the subtle differences. These computations were performed on a GPU cluster.* | |
| --- | --- |
| Model | Objective function |
| M1 | {'verbosity': 0, 'booster':'gbtree', 'objective': 'binary:logistic', 'scale_pos_weight': 3.6075131113550434,'tree_method': 'gpu_hist','eval_metrics': 'logloss', 'learning_rate': 0.09963558437961703, 'num_boost_round': 191.58714451312179, 'max-depth': 4, 'gamma': 6.140489891496016e-05, 'subsample': 0.7329970919203579, 'reg_alpha': 1.5492015574694834e-05, 'reg_lambda': 0.002889192981174229, 'colsample_bytree': 0.7341652208091972, 'min_child_weight': 0, 'n_estimators': 443} |
| M2 | {'verbosity': 0, 'booster': 'gbtree', 'objective': 'binary:logistic','scale_pos_weight': 3.6063414634146342, 'max_delta_step': 1, 'tree_method': 'gpu_hist', 'eval_metrics': 'logloss', 'learning_rate': 0.09813841335627332, 'num_boost_round': 281.7702180112571, 'max-depth': 2, 'gamma': 3.8914577758283693e-05, 'subsample': 0.7341873457611429, 'reg_alpha': 0.22771113527284387, 'reg_lambda': 1.6884029915870075e-07,  'colsample_bytree': 0.850855026540212, 'min_child_weight': 3, 'n_estimators': 473} |
| M3s | {'verbosity': 0, 'booster': 'gbtree',  'objective': 'binary:logistic', 'scale_pos_weight': 8.125875815414352, 'max_delta_step': 1, 'tree_method': 'gpu_hist', 'eval_metrics': 'logloss',  'learning_rate': 0.09880726959534625, 'num_boost_round': 198.863880711211,  'max-depth': 5, 'gamma': 1.5604867919189752e-08, 'subsample': 0.6569083845212979, 'reg_alpha': 7.461449079675969e-06, 'reg_lambda': 8.072448854162746e-08, 'colsample_bytree': 0.9103345902075206, 'min_child_weight': 0, 'n_estimators': 446} |

| M1 | 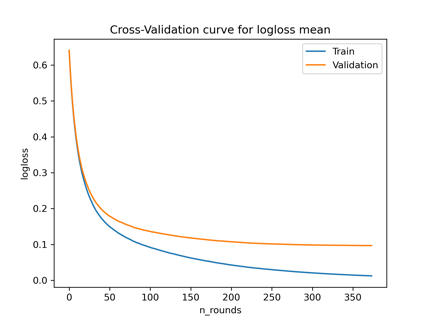 |
| --- | --- |
| M2 | 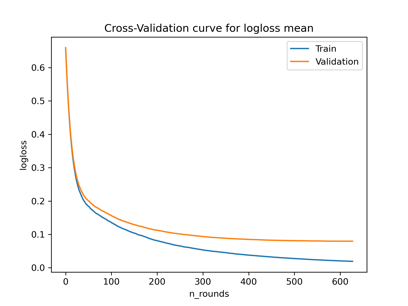 |
| M3 | 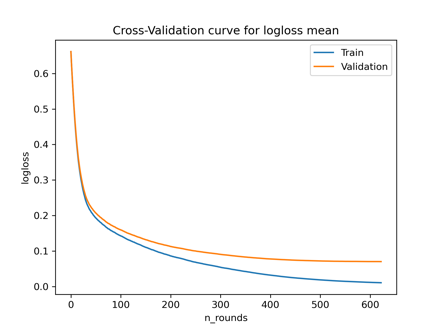 |

**Figure SI 3**: Log loss plots for the three constructed models. A convergence trend observed in the epochs shows the stability of the models.

**Key drivers of AIV outbreaks for M1 and M3**

The top global ranking features with a positive impact on M1 are the minimum temperature of the third quarter (min_temp_q3), mean temperature of the second quarter (mean_temp_q2), water index of quarter one (ndwi_q1), and high vegetation index of quarter one (lai_hv_q1). Disabling the wild bird labels In M3 resulted in a few observable differences from the M2 model. The validation and the training log loss shift signifying a reduction in the model’s performance on the test data (See Figure 2 in the main text). We also observe differences in the global ranking of SHAP values with loading being the top ranked. The min_temp_q3, unloading, ndvi_q4, ndwi_q1 and annual GDP follow respectively (Figure SI 4).

| **M1** | **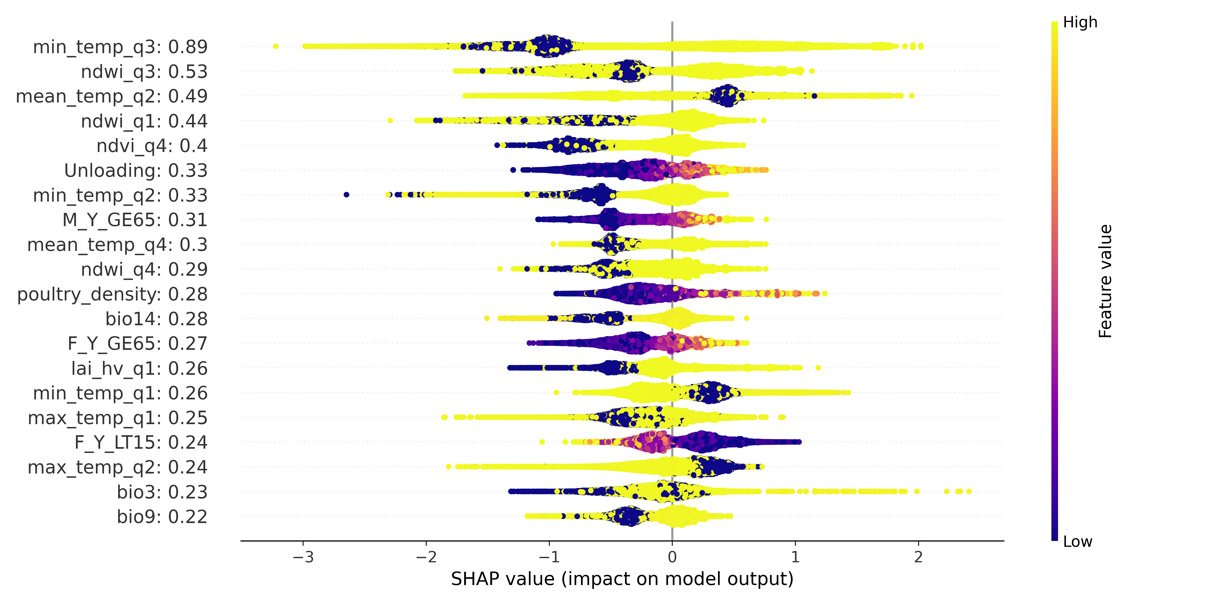** |
| --- | --- |
| **M3** | **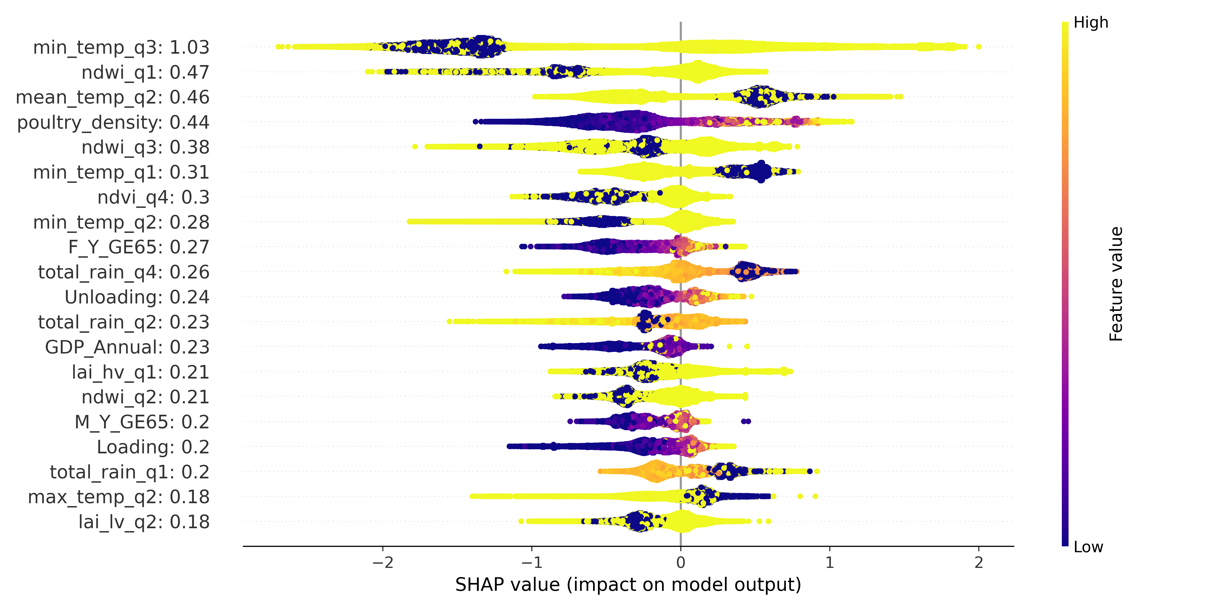** |

**Figure SI 4**: Summary SHAP plots for models M1 and M3. In M1, positive outbreaks are largely driven by climatic factors while in M3, they are driven by a combination of economic and environmental factors.

| **Table SI 4**: *Comprehensive performance report for all the models at thresholds (0.1 to 0.5). As explained in the text, M2 had a well-balanced performance when adjusting the thresholds.* | | | |
| --- | --- | --- | --- |
| Model | | % True Positives | % True Negatives |
| M1 |  |  |  |
|  | 0.1 | 98.72 | 73.58 |
|  | 0.2 | 98.40 | 77.36 |
|  | 0.3 | 97.97 | 81.51 |
|  | 0.4 | 97.76 | 83.77 |
|  | 0.5 | 97.55 | 86.79 |
| M2 |  |  |  |
|  | 0.1 | 99.05 | 72.72 |
|  | 0.2 | 98.11 | 81.34 |
|  | 0.3 | 98.11 | 86.36 |
|  | 0.4 | 98.11 | 89.58 |
|  | 0.5 | 97.64 | 90.73 |
| M3 |  |  |  |
|  | 0.1 | 99.47 | 73.26 |
|  | 0.2 | 99.36 | 79.07 |
|  | 0.3 | 99.26 | 83.33 |
|  | 0.4 | 98.94 | 86.05 |
|  | 0.5 | 98.94 | 89.53 |

**Figure SI 5**: SHAP contribution for each class/category obtained as percentages from the individual features for M1, M2, and M3 respectively.


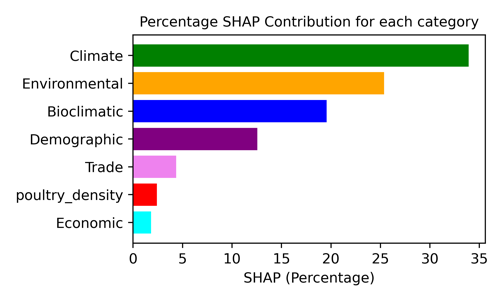

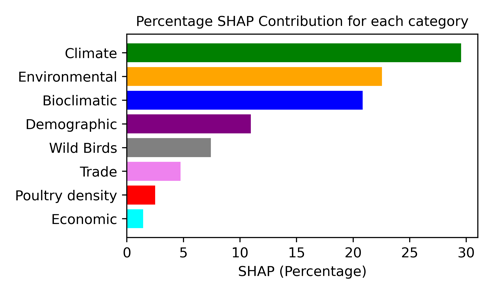

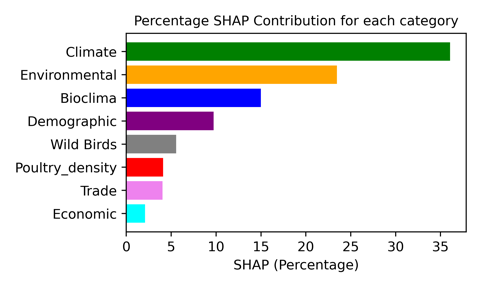

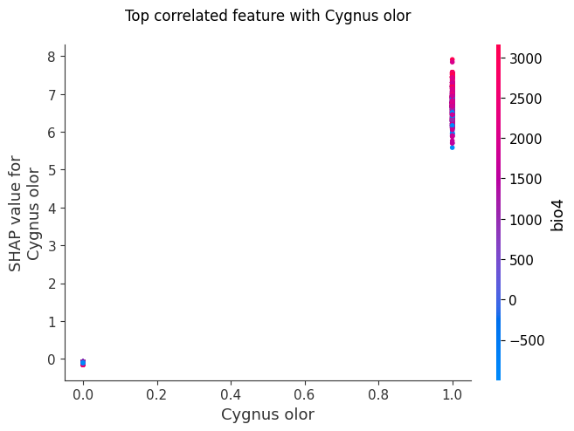

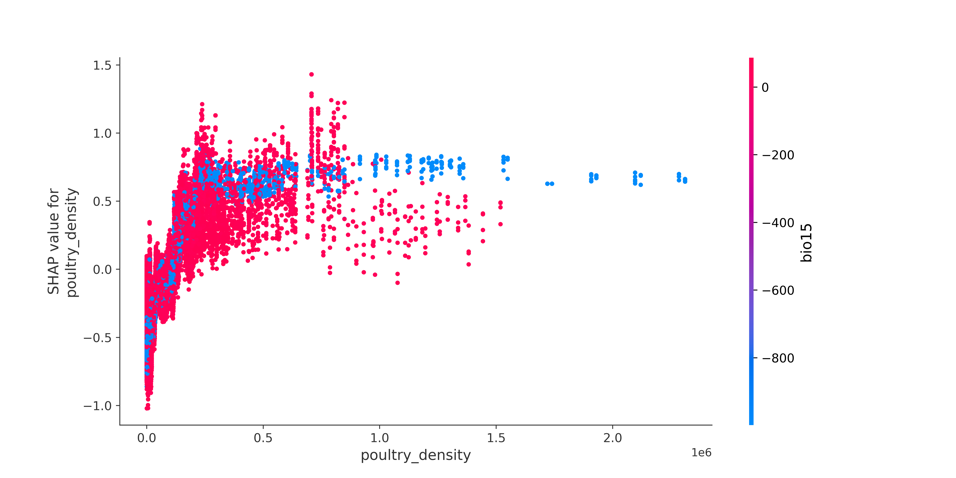

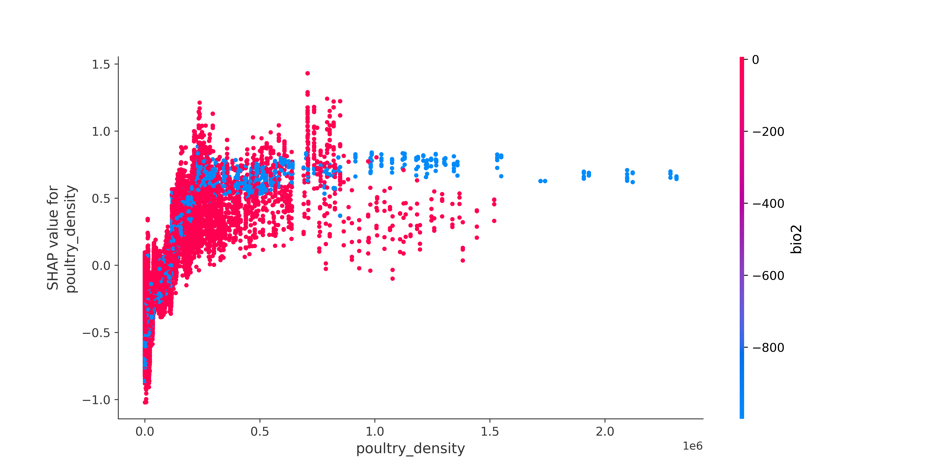


Figure SI 6: Bi-variate SHAP analysis of poultry density with bio15 and bio2. Among the wild birds, Cygnus olor interacts with bio4. The wild bird interaction is however highly local. This means that the effect is pronounces only within specific regions and not throughout Europe.


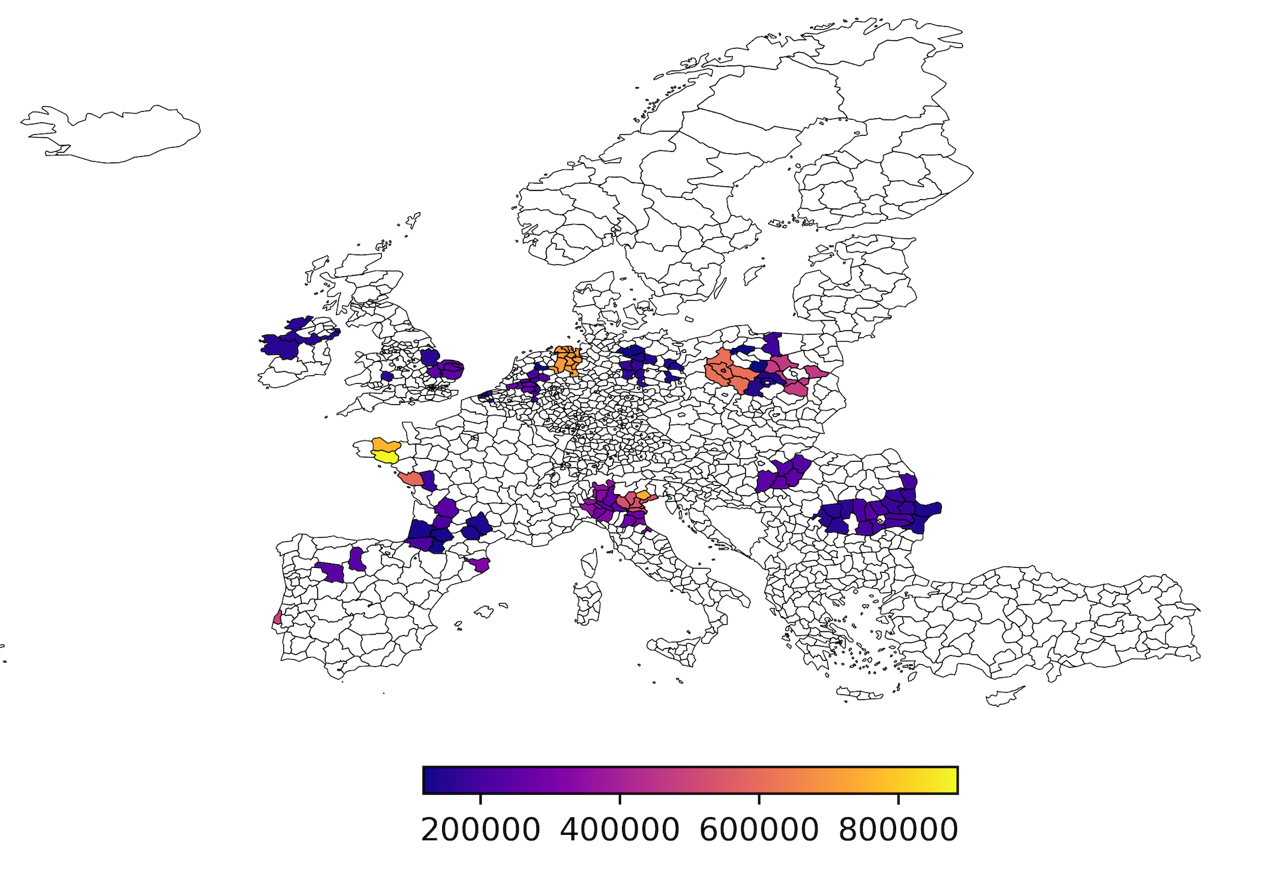

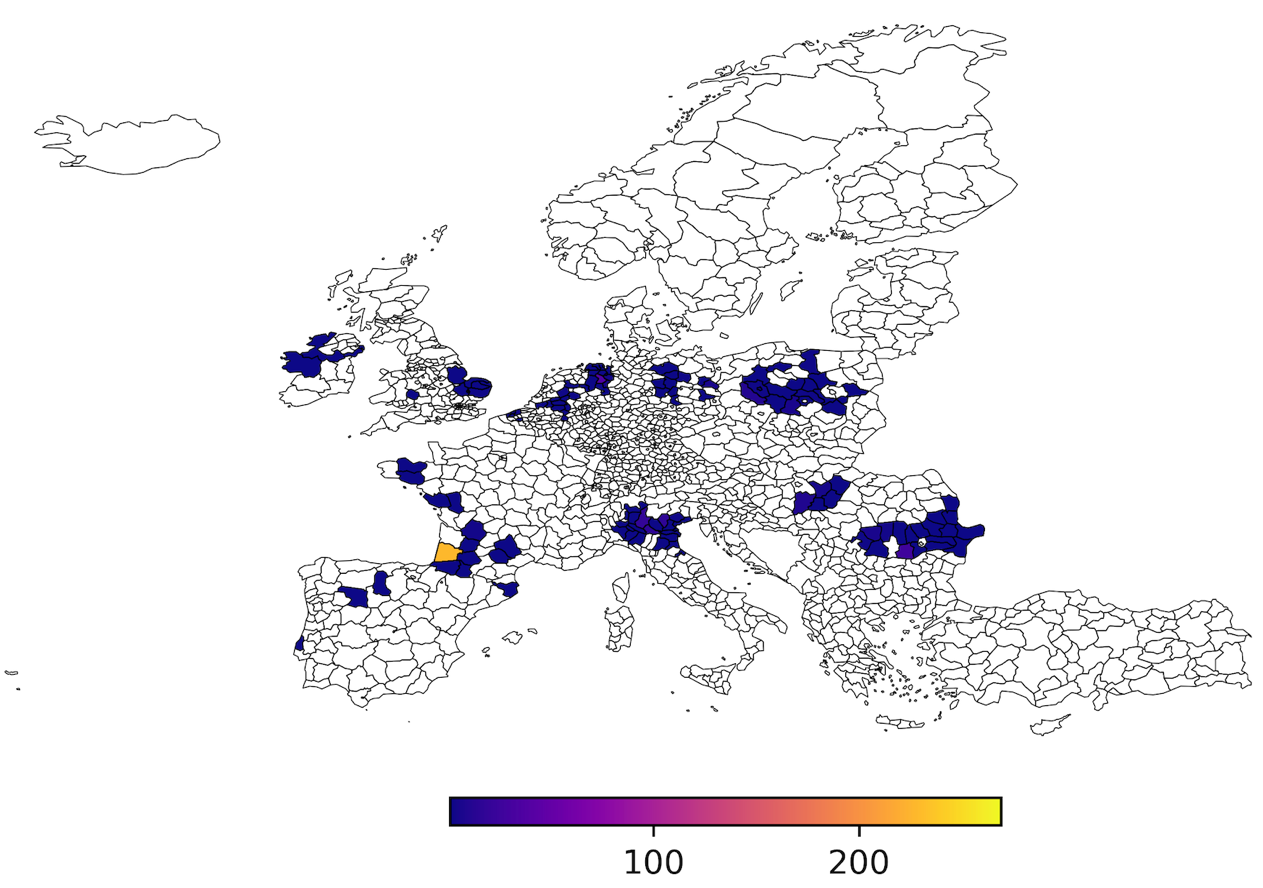


**Figure SI 7**: (**TOP**) Poultry density distribution of regions captured by SHAP as positively impacting on the model. (**BOTTOM**) Actual distribution of the regions predicted by SHAP as positively impacting the model. As is evident, the model captures regions of high density with the highest being farm holdings with over 800,000 units.

# Discussion

**Evaluation metrics**

From the evaluation plot shown in (Figure 2 main text), it is evident that the introduction of different features meaningfully impacts the predicted outcome of our models. The logloss of our model (M2) drastically reduces i.e., accuracy of prediction increases when the wild birds are used as features. A change in the thresholds confirms its sensitivity to the shifts with a difference of up to 26% (*Table SI 4*). Changing thresholds is a way of creating artificial biasness in cases where the data is imbalanced guaranteeing correct class distributions [8]. Sensitivity enables the model to capture any changes in feedback loops either through deviations in the outbreaks or outright bias in the models. In other words, our model can produce consistent results when predicting the presence of AIVs in the event of external data variation. The consistency affirms our model’s reliability to distinguish between two classes of unseen AIV outbreaks from a set of time-series dataset.

M1 served as a generic template for predicting AIV outbreaks. In quarter one, the environmental features, water index and vegetation index, positively drive the outbreaks. During cold seasons, these habitats could contribute to migration of wild birds and result in more interactions with other mammals. This finding is in line with literature where migrating birds, especially those moving over short distances, are reported to easily shed AIVs [9]. Temperature plays a role in driving AIV events during quarter two as well as quarter three. Like M2, we observe complex relationships where the mean temperature in quarter two and minimum temperature quarter three variables exhibit a U-shaped pattern (see the shifted blue and yellow plots in Figure SI 4). In this model, poultry density ranks quite low compared to M2 and M3.

Model M3 was constructed by switching off the targets of the wild birds and represents an association between wild birds with highly pathogenic AIVs and domesticated birds while they (wild birds) remain unaffected. This model is akin to the prediction of domesticated outbreaks after being infected by wild birds or simply an outbreak which occurs in the absence of wild birds. Poultry density together with the ndwi_q1, min_temp_q3, and mean_temp_q2 emerged as the most influential global feature. As seen in (Figure SI 4 M3.). Like M1 and M2 above discussed models, the min_temp_q3 is the dominant climate variable.

The introduction of poultry density as a leading variable highlights the difference between M3 and the previous two models. This influence is confirmed by the overall SHAP percentage contribution as this category ranks above the trade category (Figure SI 6). A clear implication of this model is that one would use poultry density as a main indicator whenever HPAI in poultry outbreak is reported. To an extent, such measures exist within Europe. However, active surveillance should be conducted within these farms especially when there exist anomalies in climate variables captured by the model. Interventions can be introduced early enough to mitigate the extent of impact of AIV using available data. Insights from this model introduce an opportunity to strengthen poultry surveillance policies which have been implemented in Europe.

References

[1] F. Pedregosa, G. Varoquaux, A. Gramfort, V. Michel, B. Thirion, O. Grisel, M. Blondel, P. Prettenhofer, R. Weiss, V. Dubourg, J. Vanderplas, A. Passos, D. Cournapeau, M. Brucher, M. Perrot, É. Duchesnay, Scikit-learn: Machine Learning in Python, Journal of Machine Learning Research 12 (2011) 2825–2830. http://jmlr.org/papers/v12/pedregosa11a.html (accessed August 1, 2023).

[2] Z. Farooq, J. Rocklöv, J. Wallin, N. Abiri, M.O. Sewe, H. Sjödin, J.C. Semenza, Artificial intelligence to predict West Nile virus outbreaks with eco-climatic drivers, The Lancet Regional Health – Europe 17 (2022). https://doi.org/10.1016/j.lanepe.2022.100370.

[3] T. Chen, C. Guestrin, XGBoost: A Scalable Tree Boosting System, in: Proceedings of the 22nd ACM SIGKDD International Conference on Knowledge Discovery and Data Mining, 2016: pp. 785–794. https://doi.org/10.1145/2939672.2939785.

[4] S. Raschka, V. Mirjalili, Python machine learning: machine learning and deep learning with Python, scikit-learn, and TensorFlow 2, Third edition, Packt, Birmingham Mumbai, 2019.

[5] J.H. Friedman, Greedy function approximation: A gradient boosting machine., The Annals of Statistics 29 (2001) 1189–1232. https://doi.org/10.1214/aos/1013203451.

[6] B. Vrigazova, The Proportion for Splitting Data into Training and Test Set for the Bootstrap in Classification Problems, Business Systems Research: International Journal of the Society for Advancing Innovation and Research in Economy 12 (2021) 228–242. https://hrcak.srce.hr/ojs/index.php/bsr/article/view/17347 (accessed May 4, 2024).

[7] O. Campesato, Artificial Intelligence, Machine Learning, and Deep Learning, Mercury Learning and Information, 2020.

[8] F. Provost, Machine Learning from Imbalanced Data Sets 10, (2001).

[9] V. Martin, A. Forman, J. Lubroth, Preparing for highly pathogenic avian influenza, rev. ed, Food and Agriculture Organization of the United Nations, Rome, 2009.
